# Searching for biomarkers to help distinguish Merkel cell carcinoma from cutaneous small cell lung cancer with gene expression analysis

**DOI:** 10.1111/srt.13789

**Published:** 2024-06-19

**Authors:** Joshua D. Bloomstein, Mohammad S. Doost, Summer N. Meyer, Maija Kiuru, Daniel B. Eisen

**Affiliations:** ^1^ Department of Medicine University of Washington Seattle Washington USA; ^2^ Department of Dermatology University of California Davis California USA

## 1 INTRODUCTION

Merkel cell carcinoma (MCC) is a rare, aggressive cutaneous malignancy that primarily affects the head, neck, and extremities. MCC can be difficult to distinguish from other skin cancers by morphology or dermoscopy.^1^ Hematoxylin and eosin staining of MCC shows sheets or nodules of small, round, uniform basaloid cells with scant cytoplasm, mimicking metastatic cutaneous small cell lung cancer (SCLC), basal cell carcinoma, cutaneous lymphoma, melanoma, Ewing sarcoma, neuroblastoma, and rhabdomyosarcoma.^1^ Immunohistochemistry (IHC) staining can help with diagnosis, though in certain cases it can be difficult to distinguish MCC from cutaneous SCLC. Keratin 20 (CK20) and Thyroid transcription factor‐1 (TTF‐1) are two common markers used to differentiate MCC from SCLC. It is reported that CK20 is positive in 75%^2^ or 91%^3^ of MCCs. TTF‐1 is usually negative in MCC and positive in SCLC,^2^ yet in one study, TTF‐1 was positive in 11%^3^ of MCCs. In these studies, a substantial number of MCC samples did not show typical staining of CK20 and TTF‐1.

In CK20‐negative cases where clinical suspicion for MCC remains high, additional markers are used,^1^ and studies reported sensitivity of the marker neurofilament as 75%–76%.^3^, ^4^ In certain cases, clinical correlation and additional imaging studies are needed. To search for additional markers that could simplify diagnosis, we performed gene expression analysis with existing datasets.

## 2 METHODS

Patients treated for MCC at a single academic health center over 26 years were identified in a previous study.^5^ We reviewed the IHC staining reports to evaluate CK‐20 and TTF‐1 staining in this cohort. We downloaded MCC and SCLC microarray gene expression data from the Daily et al. study.^6^ Robust Multi‐array Average (RMA) normalized expression levels were obtained from Gene Expression Omnibus (GSE50451). The previously published study did not compare MCC to SCLC. In our analysis, only high‐confidence probes were included for normalization. For each gene, the probe with the highest average expression level across samples was used. The distribution of median expression by gene showed a bimodal distribution. To eliminate lowly expressed genes, genes with expression below the nadir were filtered.

Differential expression (DE) was performed with Limma, using Benjamini‐Hochberg *p* value adjustment, producing false discovery rate (FDR). To minimize the risk of false positive results, we used a stringent threshold of log fold‐change of 5 (32‐fold‐change) of upregulation in MCC, with FDR < 0.001, giving what we termed “MCC High” genes.

The final candidate genes met three criteria: (1) MCC High (defined above), (2) high expression in all MCC samples, and (3) low or intermediate expression in all SCLC samples. We performed an analysis for association of candidate gene expression with MCC recurrence. Cancer recurrence was annotated for 16 MCC patients and no recurrence for 9. Alpha was set at *p* < 0.05, as multiple hypothesis testing was not indicated with only 4 genes tested.

Microarray data from the Harms et al. study^7^ was used to further confirm that the candidate genes were expressed in MCC. Gene expression values from 19 primary MCC tumor samples previously generated with RMA normalization and Limma in log2 format were downloaded from Gene Expression Omnibus (GSE39612) and were visualized. Statistical analyses and plots were produced with R version 4.1.1 with the package “limma” and Python version 3.6.15 with the package “seaborn.”

## 3 RESULTS

**FIGURE 1:**
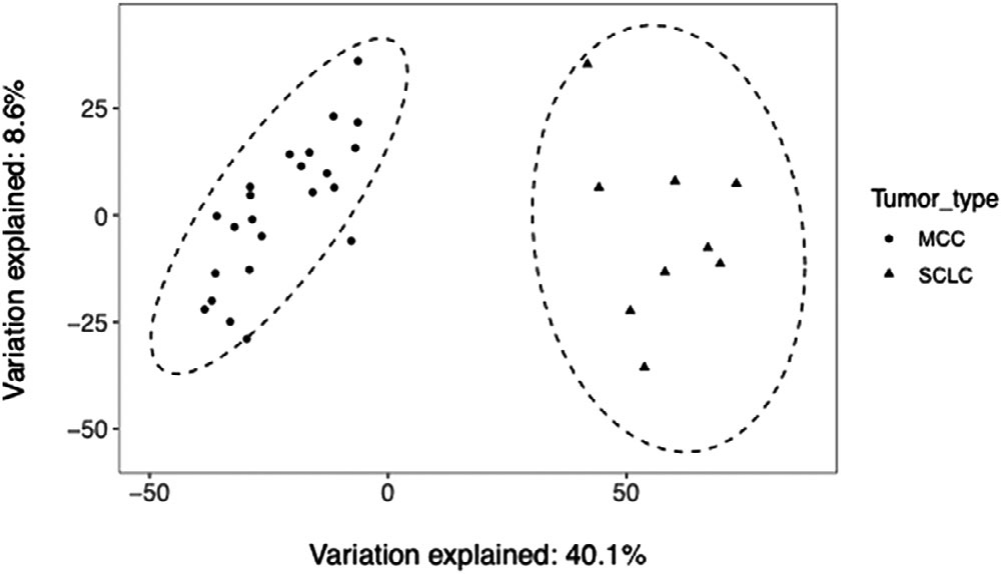
MCC versus SCC gene expression principal component analysis Principal component analysis was used to estimate global transcriptomic differences of MCC and SCLC samples.

**FIGURE 2:**
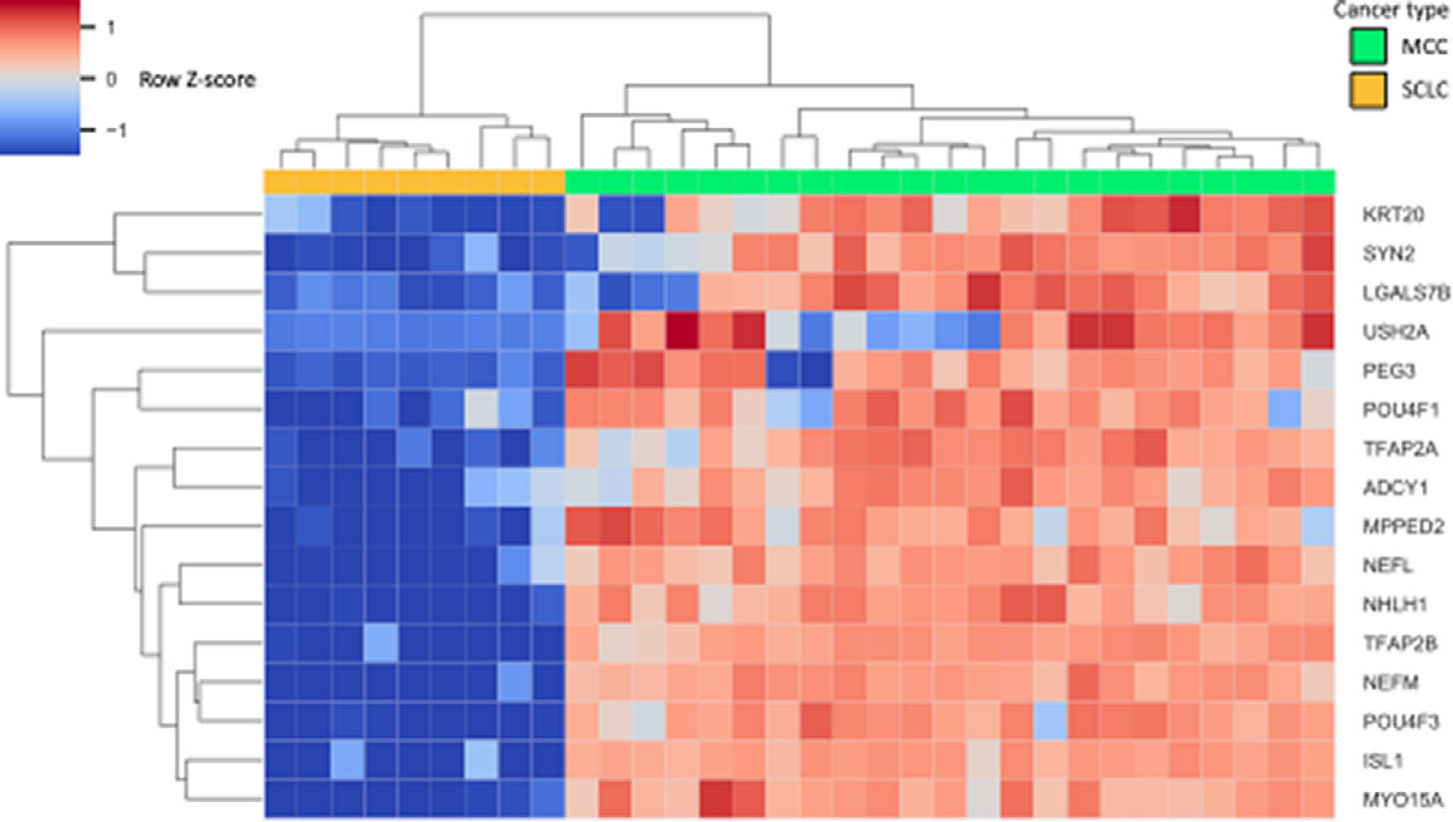
MCC High gene expression heatmap Gene expression of genes with 5 log fold‐change higher expression and FDR < 0.001 in MCC was visualized in a heatmap. Samples and genes were arranged by hierarchical clustering using Euclidean distance and average linkage. Fold‐change is expressed in linear scale as Z score across samples.

**FIGURE 3:**
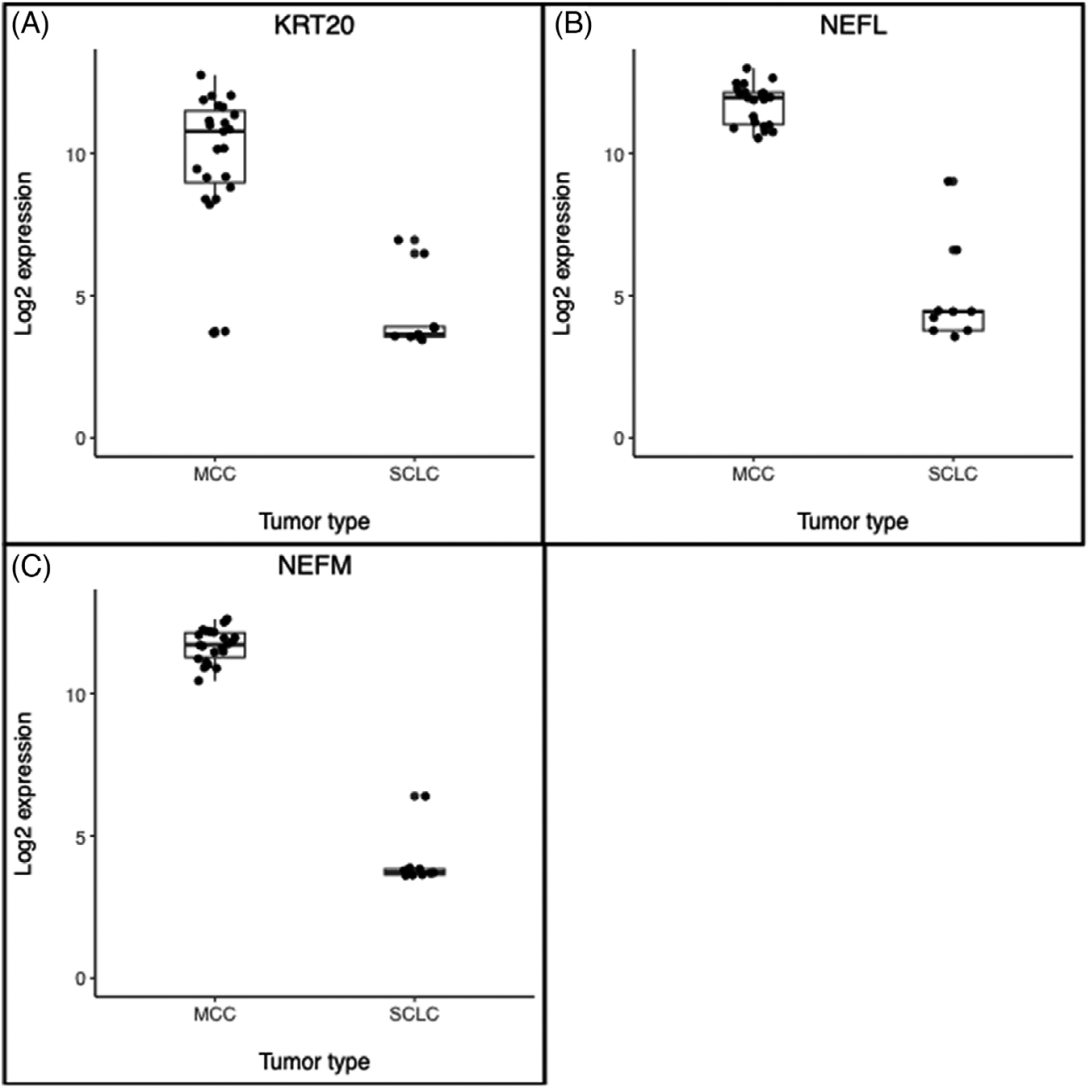
MCC versus SCLC canonical marker gene expression box dot plots Gene expression of 3 MCC High genes, KRT20 (A), NEFL (B), NEFM (C), that correspond to proteins currently used as IHC markers are shown. KRT20 was highly expressed in most MCC samples, while NEFL and NEFM were highly expressed in all MCC samples.

The cohort treated at UCD for MCC showed 91% CK20 positivity and 12% TTF‐1 positivity. In the Daily et al. cohort, principal component analysis showed separation of MCC from SCLC samples (Figure 1). 16 genes were MCC High (Table 1, Figure 2), including canonical MCC IHC genes *KRT20*, neurofilament light chain (*NEFL*), and neurofilament medium chain (*NEFM*). However, 3 of the MCC samples showed low expression of *KRT20* and 4 SCLC samples showed intermediate expression (Figure 3). *NEFL* and *NEFM* showed high expression among MCC samples and low expression among SCLC samples.

**TABLE 1:** MCC high genes DE comparison of MCC and SCLC showed that 16 genes had greater than 5 log fold‐change higher expression in MCC with FDR < 0.001.

Gene symbol	Fold‐change	*p*‐value	FDR	Gene name
NEFM	210.8	7.57E‐25	4.08E‐21	Neurofilament, medium polypeptide
TFAP2B	210.8	1.07E‐22	2.87E‐19	Transcription factor AP‐2 beta
ISL1	158.7	3.17E‐19	2.44E‐16	ISL LIM homeobox 1
NEFL	116.2	1.99E‐17	1.34E‐14	Neurofilament, light polypeptide
PEG3	76.1	2.73E‐09	4.88E‐08	Paternally expressed 3
MYO15A	73.5	7.48E‐21	8.07E‐18	Myosin XVA
MPPED2	69.6	1.17E‐14	2.26E‐12	Metallophosphoesterase domain containing 2
NHLH1	56.1	8.14E‐22	1.10E‐18	Nescient helix loop helix 1
KRT20	46.9	6.11E‐08	6.07E‐07	Keratin 20
SYN2	46.2	1.96E‐11	9.97E‐10	Synapsin II
POU4F1	42.2	1.38E‐10	4.34E‐09	POU class 4 homeobox 1
TFAP2A	37.8	1.23E‐16	4.73E‐14	Transcription factor AP‐2 alpha
LGALS7B	36.3	1.58E‐07	1.32E‐06	Lectin, galactoside‐binding, soluble, 7
POU4F3	35.3	1.88E‐19	1.69E‐16	POU class 4 homeobox 3
USH2A	32.2	6.35E‐06	3.12E‐05	Usher syndrome 2A
ADCY1	32.2	3.50E‐12	2.52E‐10	Adenylate cyclase 1

*Note*: These genes were designated as MCC high.

**FIGURE 4:**
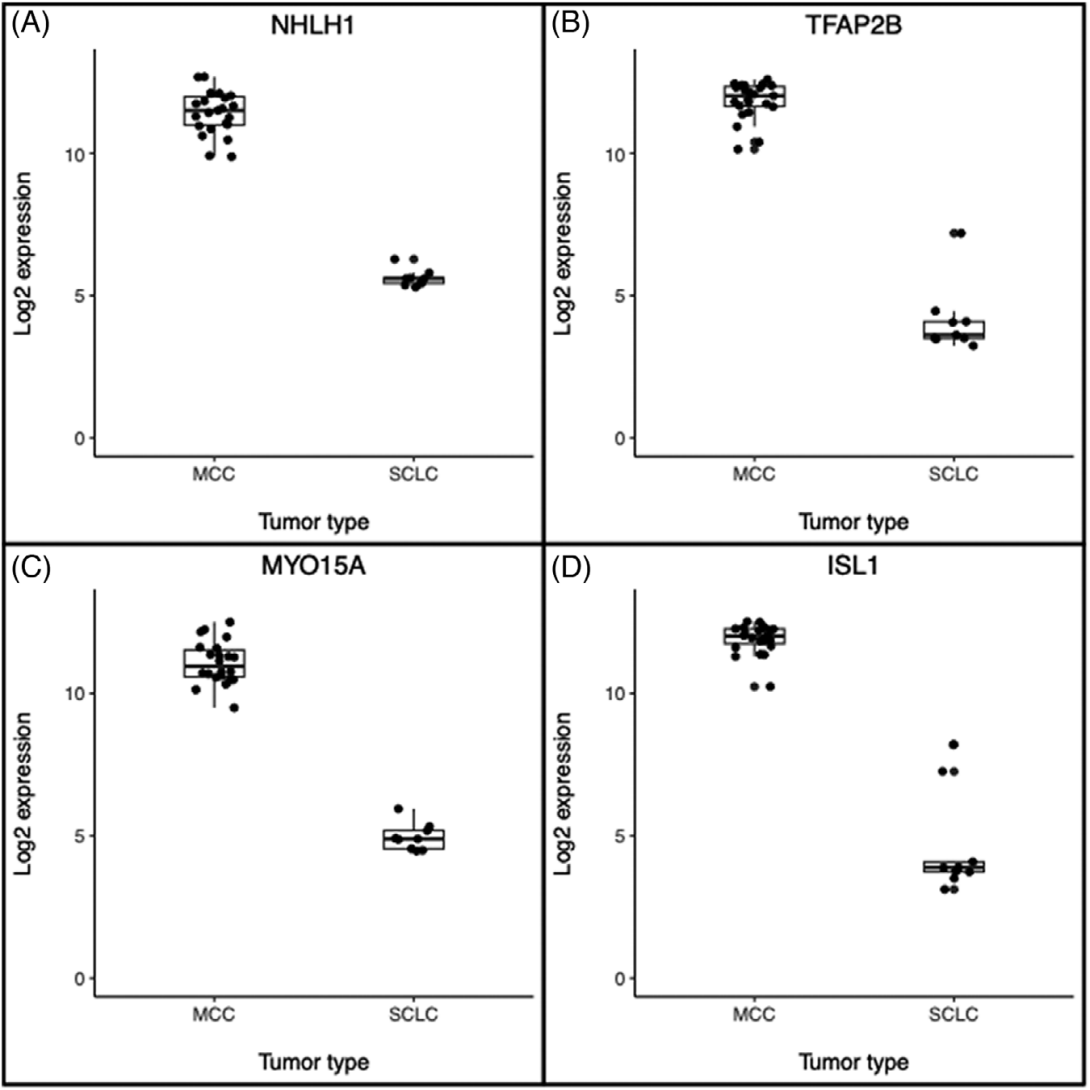
MCC versus SCLC candidate marker gene expression dot plots Gene expression of 4 MCC High candidate genes, NHLH1 (A), TFAP2B (B), MYO15A (C), ISL1 (D) is shown. Each gene showed higher expression in all MCC samples than SCLC samples. Box plots show the median, 75th and 25th percentiles, and 1.5 times the interquartile ranges.

4 MCC High genes, *NHLH1*, *MYO15A*, *ISL1*, and *TFAP2B*, met our three criteria (Figure 4). DE analysis in MCC samples showed increased expression of *NHLH1* in patients with MCC recurrence, while there was no evidence of difference among the other 3.

**FIGURE 5:**
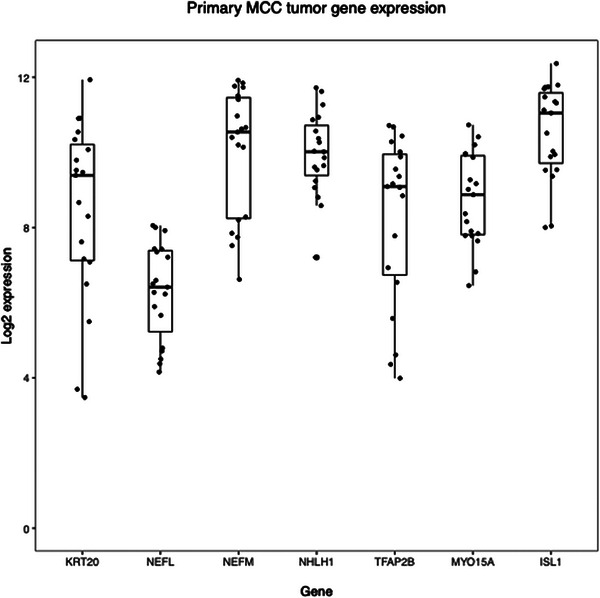
Canonical and candidate gene expression in MCC Expression of canonical and candidate marker genes was assessed using microarray data from a second cohort of patients with MCC. All candidate genes were expressed in the second cohort. Box plots show the median, 75th and 25th percentiles, and 1.5 times the interquartile ranges.

The Harms et al. microarray dataset confirmed expression of the 3 canonical genes and 4 candidate genes in MCC (Figure 5). *KRT20* showed a wide range of expression, while *NHLH1* and *MYO15A*, and *ISL1* showed a tighter range of high expression, suggesting the 3 candidate genes have reliably higher expression.

## 4 DISCUSSION

Review of IHC of patients treated for MCC at UCD between 1994 and 2020 showed not all were positive for CK‐20 or negative for TTF‐1, similar to previous studies, suggesting the markers are imperfect. Our DE analysis showed that *NHLH1*, *MYO15A*, *ISL1*, and *TFAP2B* were highly expressed in all MCC tumors and lowly expressed in all SCLC tumors in another cohort. These genes showed greater upregulation in MCC than *KRT20* (corresponding to CK‐20). A previous study, however, found that ISLI IHC staining is strongly positive in both MCC and SCLC,^8^ suggesting this is not a good marker. *NHLH1, MYO15A, and TFAP2B* may be worthwhile to study further on the RNA and protein levels to see if they can be used for IHC to distinguish MCC from SCLC.

To our knowledge, this is the only gene expression dataset that has both MCC and SCLC, leading to small sample size. Inherent differences between gene expression datasets are not easily or reliably reconciled, precluding dataset combination. Microarray technology is inferior to RNA sequencing (RNA‐seq), yet to date there is no RNA‐seq dataset with these two cancers.

A larger RNA‐seq study of MCC and SCLC could provide more insight into transcriptomic differences. If the expression differences we found are validated, then the corresponding proteins could be studied in‐depth. Ideally, new markers could facilitate definitive diagnosis of MCC in borderline cases.

## Data Availability

The datasets we re‐analyzed that were produced in the previous studies are available in the Gene Expression Omnibus repository under accession numbers GSE50451 (https://www.ncbi.nlm.nih.gov/geo/query/acc.cgi?acc=GSE50451) and GSE39612 (https://www.ncbi.nlm.nih.gov/geo/query/acc.cgi?acc=GSE39612).
